# Implications of Systemic Inflammation and Periodontitis for Major Depression

**DOI:** 10.3389/fnins.2018.00483

**Published:** 2018-07-18

**Authors:** Sadayuki Hashioka, Ken Inoue, Maiko Hayashida, Rei Wake, Arata Oh-Nishi, Tsuyoshi Miyaoka

**Affiliations:** ^1^Department of Psychiatry, Shimane University, Izumo, Japan; ^2^Health Service Center, Kochi University, Kochi, Japan

**Keywords:** major depression, systemic inflammation, periodontitis, pro-inflammatory cytokines, microglia, neuroinflammation

## Abstract

Increasing evidence suggests that infection and persistent low-grade inflammation in peripheral tissues are important pathogenic factors in major depression. Major depression is frequently comorbid with systemic inflammatory diseases/conditions such as rheumatoid arthritis, allergies of different types, multiple sclerosis, cardiovascular disease, inflammatory bowel disease, chronic liver disease, diabetes, and cancer, in which pro-inflammatory cytokines are overexpressed. A number of animal studies demonstrate that systemic inflammation induced by peripheral administration of lipopolysaccharide increases the expression of pro-inflammatory cytokines in both the periphery and brain and causes abnormal behavior similar to major depression. Systemic inflammation can cause an increase in CNS levels of pro-inflammatory cytokines associated with glial activation, namely, neuroinflammation, through several postulated pathways. Such neuroinflammation can in turn induce depressive moods and behavioral changes by affecting brain functions relevant to major depression, especially neurotransmitter metabolism. Although various clinical studies imply a causal relationship between periodontitis, which is one of the most common chronic inflammatory disorders in adults, and major depression, the notion that periodontitis is a risk factor for major depression is still unproven. Additional population-based cohort studies or prospective clinical studies on the relationship between periodontitis and major depression are needed to substantiate the causal link of periodontitis to major depression. If such a link is established, periodontitis may be a modifiable risk factor for major depression by simple preventive oral treatment.

## Introduction

It has been well documented that inflammation is closely related to major depression, even though it remains uncertain whether inflammation is a cause or a result of the mental illness. The association between inflammation and major depression has been supported by the well-known clinical observation that pro-inflammatory cytokines such as interferon (IFN)-α, which is used to treat hepatitis C, renal cancer, and multiple myeloma, and interleukin (IL)-2, which is used to treat renal cancer and angiosarcoma, frequently induce depressive symptoms as side effects (Hashioka et al., 2009). More than 50% of patients treated with high dose IFN-α met the diagnostic criteria for major depression within 3 months after administration of IFN-α (Musselman et al., 2001). Moreover, physically ill patients with chronic inflammation often manifest symptoms of major depression (Dantzer, 2017). These clinical observations parallel findings are observed in a number of animal studies. Peripheral administration of endotoxin lipopolysaccharide (LPS), a component of Gram-negative bacterial cell wall, into mice has been shown to increase expression of pro-inflammatory cytokines, such as IL-1β, IL-6, and tumor necrosis factor (TNF)-α, in both the periphery and brain, especially in microglia and perivascular macrophages in the brain (Layé et al., 1994; Godbout et al., 2008), and cause abnormal behavior similar to major depression (O’Connor et al., 2009; Biesmans et al., 2013). It has also been demonstrated that systemic administration of pro-inflammatory cytokines induces depressed behavior in rodents (Anforth et al., 1998; Kaster et al., 2012).

Major depression seems to be a heterogeneous disease with different genetic and environmental factors contributing to the disease development (Shelton, 2007). Systemic infection and inflammatory processes may also play a role in the pathogenesis of major depression, at least in a subset of susceptible individuals. The aim of this article is to review the possible role of systemic inflammation in the pathogenesis of major depression and the possible causal link of periodontitis, which is one of the most common inflammatory diseases in adults, to the clinical onset and development of major depression.

## Systemic Inflammation and Major Depression

Major depression is frequently comorbid with systemic inflammatory diseases/conditions such as rheumatoid arthritis, allergies of different types, multiple sclerosis, cardiovascular disease, inflammatory bowel disease, chronic liver disease, diabetes, and cancer, in which pro-inflammatory cytokines are overexpressed (Evans et al., 2005; Leonard, 2007; D’Mello and Swain, 2017). For example, patients with major depression are twice as likely to develop heart disease after controlling for effects of smoking and hypertension (Anda et al., 1993). Therefore, persistent low-grade inflammation in peripheral tissues may be a common mechanism underlying the high comorbidity between major depression and physical illness.

Emerging evidence indicates that systemic inflammation has a profound impact on behavior. It is well established that systemic inflammation causes the synthesis of pro-inflammatory cytokines, which communicate with the brain to induce the spectrum of behavioral changes known as sickness behavior (Perry, 2004). Sickness behavior includes certain signs similar to clinically relevant symptoms of major depression, such as appetite loss, sleep disturbance, reduced activity, and reduced social interest. However, some neuroscientists suppose that sickness behavior is rather an adaptive response to infection by pathogens and fully reversible once the pathogen cleared; this is not a case for major depression (Dantzer et al., 2008). They have established a murine model of inflammation-induced depression using systemic administration of LPS and claim that the LPS-induced depressive-like behavior can be distinguished from sickness behavior in the animal model (Dantzer et al., 2008; O’Connor et al., 2009). Specifically, sickness behavior measured by reduced food intake, decreased social exploration, and reduced locomotor activity develop within a few hours after LPS injection and wanes off by 12–16 h post LPS. Subsequently, depressive-like behavior occurs 24 h after LPS injection as shown by prolonged immobility time in the forced swimming test (FST), the tail suspension test (TST), and the reduced sucrose preference. These authors also insist that this dissociation between sickness behavior and depressive-like behavior in response to LPS is consistent with the time course of the development of symptoms of depression in response to IFN-α in patients with cancer (Dantzer et al., 2008; Dantzer, 2017). In this time course, neurovegetative symptoms, including reduced appetite, sleep disorders, and fatigue, emerge first in response to repeated injections of IFN-α, whereas mood and cognitive symptoms emerge later (Capuron et al., 2002). The experimental finding of mice that depressive-like behavior is present without the confounding effects of sickness behavior 24 h after systemic LPS injection is, however, controversial. Zhu et al. (2010) have shown that depressive-like behavior appears even at earlier time points (e.g., at 10 min – a few hours after LPS injection) and others have shown that mice receiving high dose of LPS still exhibit marked sickness behavior 24 h after LPS injection (Berg et al., 2004; Godbout et al., 2008; Biesmans et al., 2013). This inconsistency may stem from difference in the age of mice used, the LPS dose, the LPS serotype, the application route, and the assays used. It has been demonstrated that selective serotonin reuptake inhibitor and serotonin norepinephrine reuptake inhibitor attenuate LPS-induced depressive-like behavior and LPS-induced increase in serum levels of pro-inflammatory cytokines in mice (Ohgi et al., 2013).

The animal studies mentioned above employed single systemic administration of LPS, which can cause acute and strong but ephemeral activation of peripheral immune system. However, inflammation-associated depression in human is rather linked to chronic inflammation (Leonard, 2018). Recently, there have been several animal studies which aim to establish attractive translational models for inflammation-associated depression in humans. Kubera et al. (2013) have established a mouse model in which repeated and intermittent LPS administration for 4 months causes a chronic state of anhedonia, indicating that chronic LPS administration might be a more relevant approach to induce depressive-like behavior. Moreau et al. (2005, 2008) have shown that inoculation of Bacillus Calmette-Guerin (BCG), an attenuated form of *Mycobacterium bovis*, into mice elicits a prolonged increase in serum levels of the pro-inflammatory cytokine IFN-α for 26 days, and the initial episode of sickness behavior, which develops in a few days after BCG inoculation, is followed by a long-lasting decrease in sucrose preference and an increase of immobility in the FST and the TST.

## How Does Systemic Inflammation Affect Moods and Behavior?

How the periphery communicates with the brain, modulates neurotransmission, and consequently changes behavior is not entirely clear. Nevertheless, several routes of communication between the systemic immune system and the brain have been postulated (Perry, 2004; Capuron and Miller, 2011; D’Mello and Swain, 2017). A systemic inflammatory response results in the secretion of pro-inflammatory cytokines, including IL-1β, IL-6, and TNF-α, from activated macrophages and monocytes. The pro-inflammatory cytokines circulate in the blood and communicate with neurons and microglia in the brain via the communication pathways mentioned below. Any of these pathways could ultimately lead to an increase of pro-inflammatory cytokines in the brain and microglial activation (hereinafter, referred to as neuroinflammation in this article). The first route is the neural pathway through which systemic cytokines directly activate primary afferent nerves, such as the vagus nerve. The signal reaches the primary and secondary projection of the neural pathway reaching first the nucleus tractus solitaries and subsequently various hypothalamic brain nuclei (Capuron and Miller, 2011). It has been shown that subdiaphragmatic vagotomy blocks the LPS-induced sickness behavior in rats (Bluthé et al., 1994), while it does not affect the LPS-induced synthesis of pro-inflammatory cytokines at the periphery. The second route is the humoral pathway involving the choroid plexus and circumventricular organs, which lack an intact blood–brain barrier (BBB). These leaky regions may be access points for circulating pro-inflammatory cytokines to enter into the cerebral parenchyma by volume diffusion and elicit downstream signaling events important in altering brain function (D’Mello and Swain, 2017). The third route is cellular pathway. Systemic inflammation is associated with activation of cerebral endothelial cells (CECs) as well as an increase in circulating monocytes (D’Mello and Swain, 2017). Systemic pro-inflammatory cytokines activate CECs expressing receptors for TNF-α and IL-1β, which in turn signal to the perivascular macrophages located immediately adjacent to CECs (Perry, 2004). These perivascular macrophages subsequently communicate with microglia and thus lead to microglial activation. Activated microglia secret not only pro-inflammatory cytokines but also proteases and chemokines, including monocyte chemoattractant protein (MCP)-1. MCP-1 is supposed to be responsible for the recruitment of monocytes into the motor cortex, hippocampus, and basal ganglia regions, areas of the brain known to be involved in control of behavior (D’Mello et al., 2009).

Once the pro-inflammatory cytokine signals reach the brain from the periphery, they could affect several pathophysiological domains, functions, and neurotransmission relevant to major depression, such as anterior cingulate cortex (anxiety and arousal), basal ganglia (motivation and motor activity), neuroendocrine function (glucocorticoid resistance and altered glucocorticoid secretion), synaptic plasticity (impaired neurogenesis), and neurotransmitter metabolism (Capuron and Miller, 2011). Microglia activated by the systemic cytokine signals secret pro-inflammatory cytokines which activate indoleamine-2,3-dioxygenase (IDO), the first and rate-limiting enzyme of the kynurenine pathway. IDO catalyzes the synthesis of kynurenine from dietary tryptophan. This could contribute to depressive symptoms by reducing the availability of tryptophan required for the synthesis of serotonin and melatonin.

Microglial activation has been linked to depressive behavior. A number of animal studies have demonstrated that mice whose microglia are activated by systemic LPS injection show sickness and depressive-like behavior (Biesmans et al., 2013; Townsend et al., 2014; Norden et al., 2016). We confirmed that Gunn rats with congenital microgliosis and hyperbilirubinemia showed prolonged immobility time in both the FST and the TST, indicating that microglial activation could be related to learned helplessness (Arauchi et al., 2018). Furthermore, animal studies have shown that pharmacological inhibition of activated microglia improves both sickness behavior and depressive-like behavior. Administration of minocycline into mice reduced the LPS-induced microglial expression of pro-inflammatory cytokines and ameliorated sickness behavior and anhedonia (Henry et al., 2008). Minocycline administration completely inhibited the increase and activation of microglia in the hippocampus and reduced chronic mild stress-induced depressive-like behaviors in the FST and open field test (Wang et al., 2018). Postmortem studies also indicate that activated microglia are involved in the brain of patients with major depression (Bayer et al., 1999; Steiner et al., 2011). Iwata et al. (2016) have recently shown that psychological stress increases extracellular adenosine triphosphate (ATP) in the hippocampus. The increased extracellular ATP binds to purinergic type 2X7 receptor (P2X7R) on microglia and subsequently activates nucleotide-binding leucine-rich repeat, pyrin domain containing (NLRP) 3 inflammasome in microglia to induce microglial release of IL-1β. IL-1β has been shown to decrease neurogenesis in adult hippocampus and this effect is associated with development of anhedonia (Koo and Duman, 2008). Accordingly, the authors claim that the ATP/P2X7R-NLRP3 inflammasome cascade in microglia could be a therapeutic target for treatment of stress-related depression.

The gut-microbiota to brain route has received increasing attention for its ability to modulate brain functions. A recent animal study has shown that blockade of peripheral IL-6 receptor by anti-mouse IL-6 receptor antibody promotes rapid and sustained antidepressant action thorough normalizing the altered composition of gut microbiota in susceptible mice after social defeat stress, suggesting that gut–microbiota–brain communication has a role in robust antidepressant actions of anti-IL-6 receptor therapy (Zhang et al., 2017).

## Periodontitis and Major Depression

Periodontitis is a chronic oral multi-bacterial infection and one of the most common inflammatory disorders in adults. In 2009–2010, the total prevalence of periodontitis in adults aged 30 years and older was 47.2% in the United States (Eke et al., 2012). The World Health Organization reported that 10–15% of the world populations suffer from severe periodontitis (Petersen and Ogawa, 2005). Periodontitis can be classified as gingivitis, when the inflammation is localized in the gingival tissues, or it may assume a more severe destructive form, with the inflammatory process reaching deeper connective and bone tissue, causing bone and attachment loss, that may ultimately lead to tooth loss. Periodontitis *per se* is a low-grade chronic inflammation, but it causes or hastens the other chronic systemic inflammatory diseases, including atherosclerosis, cardiovascular diseases, diabetes, and rheumatoid arthritis (Holmstrup et al., 2017). This indicates that periodontitis is a significant source of systemic inflammatory molecules. Besides affecting the systemic inflammatory disorders, recent clinical studies imply that periodontitis is a risk factor for such a neuroinflammatory and neurodegenerative disorder as Alzheimer’s disease (AD). A preliminary study has shown a cross-sectional association between increased levels of a serologic marker of *Porphyromonas gingivalis* (*Pg*), the major pathogen of periodontitis, and impaired delayed memory and calculation among patients older than 60 years, suggesting that periodontitis is associated with cognitive impairment in the elderly (Noble et al., 2009). A 6-month observational cohort study has reported that the cognitive state of the periodontitis-present AD group signifies declined compared to that of the periodontitis-absent AD group at the follow-up point (Ide et al., 2016). Moreover, LPS from *Pg* has been detected in AD brains, but not in control brains (Poole et al., 2013).

Various clinical studies also imply a causal relationship between periodontitis and major depression (Klages et al., 2005; Rosania et al., 2009). Distress experienced by patients with periodontitis significantly correlated with the progression of periodontitis (Rai et al., 2011). Chronic stress and depression can mediate risk and progression of periodontitis through change in health-related behaviors such as oral hygiene, smoking, and diet (Warren et al., 2014). Also, as periodontitis got chronic, the occurrence of depression increased (Tang and Cao, 2011). A population-based cohort study for a long-term (10 years) follow-up period showed a higher incidence of subsequent depression in the periodontitis group (*N* = 12,708) than in the non-periodontitis group (*N* = 50832) with an adjusted hazard ratio of 1.73 when adjusted for sex, age, and comorbidity (Hsu et al., 2015). This result suggests that periodontitis is an independent risk factor for major depression regardless of sex, age, and the comorbidities except for diabetes, alcohol abuse, and cancer. Interestingly, an animal study demonstrated that tianeptine, the tricyclic antidepressants which have been shown to possess anti-inflammatory properties (Hashioka et al., 2007, 2009), reduced periodontitis severity, and ameliorated emotionality- and anxiety-related behavior in the olfactory bulbectomy rat model of depression with ligature-induced periodontitis (Breivik et al., 2006). A cross-sectional clinical study also showed that intake of fluoxetine, another type of antidepressants (i.e., selective serotonin reuptake inhibitor), is associated with lower bleeding on probing percentages and lower attachment loss in patients with chronic periodontitis and clinical depression (Bhatia et al., 2015).

## Possible Link Between Periodontitis and Development of Major Depression

Both psychosocial and biological mechanisms are supposed to link periodontitis and development of major depression. Periodontitis may increase the risk for depression through psychosocial effects of halitosis, such as shame, loneliness, embarrassment, and diminished well-being (Dumitrescu, 2016). Furthermore, edentulousness caused by periodontitis due to inflammatory destruction of tooth supporting tissues (i.e., periodontal ligament and alveolar bone) could affect the patient’s quality of life. Edentulousness impairs not only chewing, but also body image, self-esteem, and social status (Saintrain and de Souza, 2012).

The biological mechanisms by which periodontitis causes major depression are presumed to consist of two possibilities: (1) neuroinflammation (i.e., increased CNS levels of pro-inflammatory cytokines associated with glial activation) induced by systemic inflammation associated with periodontitis; (2) neuroinflammation evoked by direct invasion of periodontal pathogen and their inflammatory products into the brain. Within the periodontal pocket, bacteria exist in a stratified dental biofilm, which is made of microorganisms and their components (e.g., endotoxin LPS and virulence factors; Socransky and Haffajee, 2002). The inflamed periodontal pocket can, therefore, be a significant source of inflammatory and pathogen-derived mediators. In health, the majority of bacteria are Gram-positive aerobes. In periodontitis, approximately 85% of bacteria are Gram-negative, with the major three periodontal bacteria called the “red complex,” namely, *Tannerella forsythia*, and *Treponema denticola* and *Pg* (Kamer et al., 2008; summarized in **Table 1**). Because these bacteria are capable of invading intact pocket epithelium, periodontal bacteria and their products can gain access to the circulation (Kamer et al., 2008). This results in bacteremia and systemic dissemination of bacterial products. In fact, Gomes et al. (2018) have recently reported that subjects with chronic periodontitis and depression show highly increased root canal LPS levels compared to subjects with chronic periodontitis without depression and normal controls. This study also shows a strong positive association between chronic periodontitis or root canal LPS levels and severity of depression as measured using the Hamilton Depression Rating Scale and the Beck Depression Inventory. Therefore, increased levels of LPS are supposed to link periodontitis to subsequent depression like peripheral injection of LPS that causes depressive-like behavior in mice. Since periodontal pathogen and their products, especially LPS, can induce pro-inflammatory cytokines, such as Il-1β, IL-6, and TNF-α (Liu et al., 2017; Wu et al., 2017), these cytokines could also enter the systemic circulation. Periodontitis-induced systemic dissemination of bacteria, their LPS, and inflammatory mediators initiate systemic inflammation or deterioration if the other systemic inflammation already exists. Systemic inflammation caused by periodontitis could affect behavior and moods via possible communication pathways between the periphery and the brain leading to neuroinflammation as mentioned above.

**Table 1:** Characteristics of major Gram-negative bacteria called the “red complex” in periodontitis.

Species	Virulence factors	Neuraminidases	Related disease
*Porphyromonas gingivalis*	Lipopolysaccharide SerB protein Gingipains Hemagglutinin Fimbriae	PG0352	Rheumatoid arthritis Bacterial vaginosis

*Tannerella forsythia*	BspA Hemagglutinin	NanH (TF0035) SiaH	Atherosclerosis Bacterial vaginosis

*Treponema denticola*	CfpA	TDE0471	Bacterial vaginosis

Systemic injection of LPS has been demonstrated to break down the BBB through abnormal activation of matrix metalloproteinase (Bohatschek et al., 2001; Frister et al., 2014). Therefore, it is also presumable that pathogen and their inflammatory products can enter the brain and directly cause neuroinflammation after periodontitis-induced systemic dissemination of LPS derived from the “red complex” disrupts the BBB to some extent. Theoretically, such neuroinflammation provoked by direct invasion of periodontitis bacteria and their inflammatory products could also induce depressive moods and behavioral changes, even though there has been no postmortem study which showed the presence of LPS from the “red complex” in the brains of subjects with major depression.

## Conclusion

**FIGURE 1:**
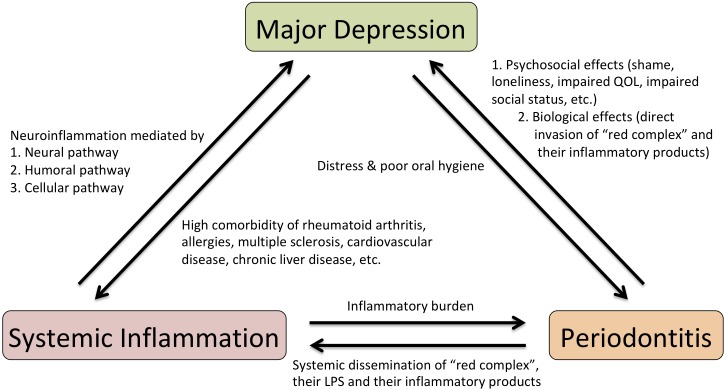
Scheme for presumed mechanisms of reciprocal connection between systemic inflammation, periodontitis and major depression. LPS, lipopolysaccharide; QOL, quality of life.

Presumed mechanisms of reciprocal connection between systemic inflammation, periodontitis, and major depression are summarized in **Figure 1**. Increasing evidence suggests that infection and persistent low-grade inflammation in peripheral tissues are important pathogenic factors that explain a possible pathophysiological mechanism of major depression. Systemic inflammation could induce depressive moods and behavioral changes by provoking neuroinflammation. Although various clinical studies imply a causal relationship between periodontitis and major depression, the notion that periodontitis is a risk factor for major depression is still a supposition. Additional population-based cohort studies or prospective clinical studies on the relationship between periodontitis and major depression are warranted to substantiate the causal link of periodontitis to major depression. If such a link is established, periodontitis may be a modifiable risk factor for major depression by simple preventive oral hygiene dealings.

## Author Contributions

SH wrote the manuscript. All authors discussed, edited, read and approved the final manuscript.

## Conflict of Interest Statement

The authors declare that the research was conducted in the absence of any commercial or financial relationships that could be construed as a potential conflict of interest.
